# Beyond the code: the role of histone methylation in cancer resistance and therapy

**DOI:** 10.1038/s41392-024-01878-1

**Published:** 2024-06-12

**Authors:** Daniel Noerenberg, Frederik Damm

**Affiliations:** 1 Charité – Universitätsmedizin Berlin, corporate member of Freie Universität Berlin, Humboldt Universität zu Berlin, and Berlin Institute of Health, Department of Hematology, Oncology, and Cancer Immunology, Berlin, Germany; 2 German Cancer Consortium (Deutsches Konsortium für Translationale Krebsforschung, DKTK), Partner Site, Berlin, Germany

**Keywords:** Haematological cancer, Haematological cancer, Cancer therapy

In a recently published paper in *Nature*, Yamagishi et al. deliver a comprehensive exploration on the mechanistic basis and subsequent emergence of resistance of targeting histone H3 lysine trimethylation (H3K27me3) using the EZH1–EZH2 dual inhibitor, valemetostat, in adult T cell leukemia/lymphoma (ATL) patients.^1^

Epigenetic variations and altered chromatin states, are common hallmarks that shape cancer development and progression. Histone methylation, a key process in the regulation of gene expression, has emerged as a crucial target in cancer therapy. Histone-modifying gene mutations are among the most frequent genetic aberrations in Hodgkin and non-Hodgkin lymphomas pointing to the central role of disrupted chromatin biology in lymphomagenesis.^2^

Histone methylation involves the addition of methyl groups to specific lysine or arginine residues on histone proteins, leading to alterations in chromatin conformation and gene expression patterns. One of the most extensively studied histone methylation inhibitors is the class of EZH2 inhibitors. EZH2 is the catalytic subunit of the polycomb repressive complex 2 (PRC2), which catalyzes the trimethylation of histone H3 at lysine 27 (H3K27me3), a repressive chromatin mark associated with transcriptional silencing. Inhibition of EZH2 has shown promising results particularly in lymphomas harboring gain-of-function mutations.^3^ However, except for *SMARCB1* deficient epitheloid sarcomas, targeting EZH2 has yet failed to demonstrate relevant clinical activity in solid malignancies.^4^

The study by Yamagishi et al. initiated by demonstrating valemetostat’s significant impact on tumor reduction and sustained clinical responses in ATL, a malignancy characterized by high relapse rates and poor prognosis. Through integrative single-cell analyses, the authors illustrate that inhibition of EZH1/2 by valemetostat disrupts the highly condensed chromatin structure mediated by H3K27me3, reinstating the expression of tumor suppressor genes, and neutralizing oncogenic pathways including depletion of malignant clones with poor prognostic variations, such as *PRKCB*, *TP53*, *IRF4*, and *PD-L1*.

**Fig. 1:**
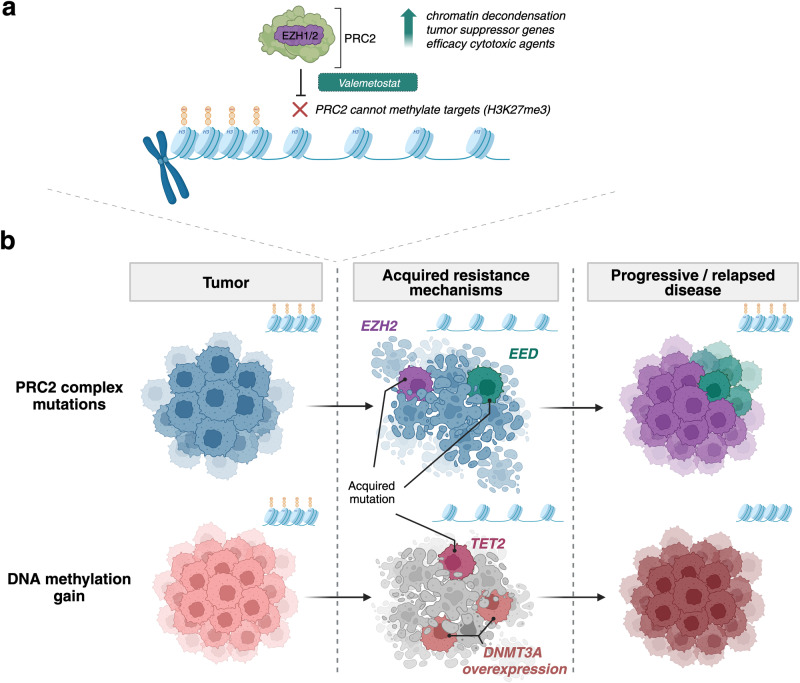
Schematic illustration of mode of action (**a**) and resistance mechanisms (**b**) in histone methylation-targeted therapy with valemetostat in adult T-cell leukemia/lymphoma (ATL). **a** By inhibiting polycomb repressive complex 2 (PRC2) via inhibition of its central enzymatic component EZH1/2, valemetostat leads to chromatin decondensation by blocking one of PRC2’s central function of H3K27 tri-methylation. This leads to upregulation of tumor suppressor genes in lymphoma cells, inhibition of tumor growth and promotion of cell death as well as depletion of malignant clones with poor prognostic variations in ATL cells. **b** Resistance to valemetostat primarily arises from two mechanisms: first, through the acquisition of mutations at the PRC2-compound interface (*EZH2* and *EED*), leading to development of resistant clones with increased H3K27me3 and condensed chromatin; second, via alterations in DNA methylation pathways, such as *TET2* mutations or increased *DNMT3A* expression, which partially poses a non-genetic mechanism of resistance evolution with condensed chromatin but without increased H3K27me3. Created with BioRender.com

Despite their clinical efficaciousness and observed durable remissions following valemetostat monotherapy, disease relapses were common.^1^ What are the mechanisms for relapse following treatment with drugs such as valemetostat? The study’s pivotal contribution lies in deciphering the mechanisms through which tumor cells develop resistance. Resistant clones, mirroring the pre-dose chromatin architecture with increased H3K27me3 expression, emerge due to acquired mutations at the PRC2-compound interface. Accordingly, among ten patients, five developed recurrent PRC2 mutations, predominantly affecting *EZH2* residues around the drug’s binding pocket at positions Y111 and Y661. More importantly, via enzymatic methylation sequencing (EM-seq) and single-cell multi-ome analysis the authors further elucidate, that in the absence of such mutations, alterations in DNA methylation pathways, particularly involving the acquisition of *TET2* mutations or increased expression levels of *DNMT3A*, represent a non-genetic mechanism of resistance evolution that poses a challenge to the treatment with valemetostat (Fig. 1).

By using single-cell RNA sequencing (scRNA-seq) and gene set enrichment analysis, the study identifies subpopulations with distinct metabolic and transcriptional characteristics. Specifically, a distinct pre-existing subpopulation characterized by high expression levels of oxidative phosphorylation (OXPHOS) genes expanded over time and led to relapse through acquisition of resistance mutations.

What makes this mechanistic research so important? Firstly, because this landmark study offers fundamental insights into histone methylation-targeted therapies, here showcased through valemetostat, and their mode of action in certain cancers like ATL. Secondly, it reveals the challenges posed by the development of resistance, mediated via genetic and epigenetic mechanisms, and highlights the dynamic interplay between therapeutic action and cancer’s adaptability to targeted therapies beyond genetic alterations.

Research on histone methylation in cancer therapy is evolving rapidly, with a focus on understanding the complex interplay between epigenetic modifications and tumor progression. Future directions include multi-omic, artificial intelligence, and hypothesis-driven multidimensional approaches for the development of combination therapies involving epigenetic drugs to enhance therapeutic efficacy and to circumvent drug resistance.

As such, combinatorial strategies aimed at targeting multiple components of the epigenetic machinery, exploiting synthetic lethal interactions or combination with immune checkpoint blockade hold promise for overcoming resistance to histone methylation-targeted therapies and vice versa.^5^ However, clinical translation of some promising preclinical studies aiming for such combinatory regimens has shown less promising results in the first clinical trials.

More importantly, developing predictive biomarkers to identify patients who are likely to benefit from histone methylation-targeted therapies and to monitor treatment response is crucial for optimizing patient selection and treatment outcomes. Dynamic assessment of biomarkers such as H3K27me3 levels, biologically relevant genetic mutations, gene expression profiles, and chromatin accessibility patterns may aid in stratifying patients based on their likelihood of response and guiding personalized treatment decisions for ATL and other malignancies characterized by aberrant histone methylation.

The paper meticulously documents the clinical advantages of EZH1/2 inhibition, the restoration of the epigenome by valemetostat, and the emergence of acquired resistance through both genetic (PRC2 mutations) and epigenetic (DNA methylation changes) alterations. It highlights the significance of understanding the interplay between epigenetic modifications and tumor biology, offering pivotal insights into potential strategies to overcome resistance and improve therapeutic outcomes.
